# Circulating SARS-CoV-2 variants in Italy, October 2020–March 2021

**DOI:** 10.1186/s12985-021-01638-5

**Published:** 2021-08-14

**Authors:** Alessia Lai, Annalisa Bergna, Stefano Menzo, Gianguglielmo Zehender, Sara Caucci, Valeria Ghisetti, Francesca Rizzo, Fabrizio Maggi, Francesco Cerutti, Giorgio Giurato, Alessandro Weisz, Chiara Turchi, Bianca Bruzzone, Francesca Ceccherini Silberstein, Nicola Clementi, Annapaola Callegaro, Fabio Sagradi, Daniela Francisci, Emmanuele Venanzi Rullo, Ilaria Vicenti, Massimo Clementi, Massimo Galli, collaborative group SCIRE SARS-CoV-2 Italian Research Enterprise, Claudia Balotta, Maria Gori, Patrizia Bagnarelli, Andreina Baj, Federica Novazzi, Andrea Orsi, Patrizia Caligiuri, Simona Boccotti, Maria Concetta Bellocchi, Loredana Sarmati, Massimo Andreoni, Nicasio Mancini, Elena Criscuolo, Rosa Gallitelli, Sophie Testa, Filippo Dragoni, Maurizio Zazzi

**Affiliations:** 1 Department of Biomedical and Clinical Sciences Luigi Sacco, University of Milan, Via G.B. Grassi 74, 20157 Milan, Italy; 2 Department of Biomedical Sciences and Public Health, Virology Unit, Polytechnic University of Marche, Ancona, Italy; 3 Microbiology and Molecular Biology Laboratory, “Amedeo di Savoia” Hospital, Turin, Italy; 4 Department of Medicine, Surgery and Dentistry ‘Scuola Medica Salernitana’ and Laboratory of Molecular Medicine and Genomics, University of Salerno, Baronissi, Italy; 5 Laboratory of Microbiology, ASST Sette Laghi, Varese, Italy; 6 Department of Medicine and Surgery, University of Insubria, Varese, Italy; 7 Genome Research Center for Health, Baronissi, Italy; 8 Hygiene Unit, San Martino Policlinico Hospital - IRCCS for Oncology and Neurosciences, Genoa, Italy; 9 Department of Experimental Medicine, University of Rome “Tor Vergata”, Rome, Italy; 10 Laboratory of Microbiology and Virology, Università “Vita-Salute” San Raffaele, Milan, Italy; 11 Biobank Unit and Microbiology and Virology Laboratory, ASST Papa Giovanni XXIII, Bergamo, Italy; 12 Haemostasis and Thrombosis Center, A.S.S.T. Cremona, Cremona, Italy; 13 Department of Medicine and Surgery, Clinic of Infectious Diseases, “Santa Maria della Misericordia” Hospital, Università degli Studi di Perugia, Perugia, Italy; 14 Department of Clinical and Experimental Medicine, Unit of Infectious Diseases, University of Messina, Messina, Italy; 15 Department of Medical Biotechnologies, University of Siena, Siena, Italy

**Keywords:** SARS-CoV-2 virus, Complete genome sequencing, COVID-19 RT-PCR testing, Spike protein, Viral variants

## Abstract

A growing number of emerging SARS-CoV-2 variants is being identified worldwide, potentially impacting the effectiveness of current vaccines. We report the data obtained in several Italian regions involved in the SARS-CoV-2 variant monitoring from the beginning of the epidemic and spanning the period from October 2020 to March 2021.

## Background

Starting from April 2020 when the Cluster 5 [1] variant was first described, multiple SARS-CoV-2 (Severe acute respiratory syndrome coronavirus 2) variants have emerged in different parts of the world. The Alpha variant (formerly known as B.1.1.7) was first identified in the UK from a sample obtained in late September 2020 [2, 3] (https://www.who.int/en/activities/tracking-SARS-CoV-2-variants/#:~:text=Naming%20SARS%2DCoV%2D2%20variants,scientists%20and%20in%20scientific%20research), Beta variant (B.1.351) was identified in October 2020 in South Africa [4], and Gamma variant (P.1) was identified in Brazil in December 2020 [5]. These variant strains show multiple changes (deletions and substitutions) in the spike protein (9 in B.1.1.7, 10 in B.1.351, and 12 in P.1) compared with the reference genome Wuhan-Hu-1 sequence (EPI_ISL_406800), some of which affect the receptor-binding domain (RBD) region. The major issue with these variants is their potential to outcompete and rapidly replace formerly prevalent lineages, first in the areas where they likely emerged and subsequently spread in many other countries [6–8]. Increased SARS-CoV-2 diversity raises the concern of escape from pre-existing immunity, elicited either by previous infection or by vaccination.

## Main text

Here, we report data from several Italian centers located in Campania, Lazio, Lombardy, Liguria, Marche, Piedmont, Tuscany, Sicily and Umbria, involved in the SARS-CoV-2 variant monitoring from the beginning of the epidemic and spanning the period from October 2020 to March 2021.

Globally, we analysed data from 3744 samples obtained by different techniques: RT-PCR variant screening assays (n = 2095), spike Sanger or Next Generation Sequencing (n = 649) and Whole Genome Sequencing (WGS; n = 1000) (Table 1). Data for the analyses were obtained from each center as a part of routine test of variant monitoring or for research purpose for those not involved in national surveillance but included in the SCIRE (SARS-CoV-2 Italian Research Enterprise) collaborative group. SARS-CoV-2 RNA positive swabs were collected from the respiratory tract of individuals who were either hospitalized or tested within screening programs. SARS-CoV-2 RNA was extracted using different commercial kits such as the Kit QIAsymphony DSP Virus/Pathogen Midi kit on the QIAsymphony automated platform (QIAGEN, Hilden, Germany), the NucleoMag 96 Virus (Macherey-Nagel, Dueren, Germany) on automated KingFisherTM ml Magnetic Particle Processors (Thermo Fisher Scientific, Waltham, MA, USA) and manually with QIAamp Viral RNA Mini Kit (QIAGEN, Hilden, Germany). RT-PCR variant screening assays were performed using TaqPath COVID-19 test (Thermo Fisher Scientific, USA), COVID-19 variant Catcher kit (Clonit srl, Milan, Italy), Allplex SARS-CoV-2 Variants (Arrow Diagnostics srl, Genoa, Italy) or multiplexed RT-qPCR developed by English consortium (https://www.protocols.io/view/multiplexed-rt-qpcr-to-screen-for-sars-cov-2-b-1-1-br9vm966?version_warning=no).

**Table 1:** Summary of analyzed data

Month	WGS (n = 1000)	Spike (n = 649)	Real time (n = 2095)	Total (n = 3744)
October 2020	137	20	1	158
November 2020	168	31	0	199
December 2020	139	34	0	173
January 2021	291	86	388	765
February 2021	193	214	827	1234
March 2021	72	264	879	1215

*WGS* whole genome sequencing

Spike sequences were obtained using home-made protocols. Full genome sequences were obtained with different protocols, by a modified version of Artic Protocol (https://artic.network/ncov-2019) using Illumina DNA Prep and IDT ILMN DNA/RNA Index kit (Illumina), by Ion AmpliSeq SARS-CoV-2 Research Panel (Thermo Fisher Scientific, Waltham, Massachusetts, USA) or by CleanPlex® SARS-CoV-2 Panel (Paragon Genomics Inc, Hayward, CA, USA). Sequencing was performed on Illumina Miseq or Nextseq platforms for all samples except for those from Marche that were sequenced with Ion GeneStudioTMS5 System instrument. The results were mapped and aligned to the reference genome obtained from GISAID (https://www.gisaid.org/, accession ID: EPI_ISL_406800) using Geneious Prime software v. 9.1.5 (Biomatters, Auckland, New Zealand) (http://www.geneious.com) or Torrent Suite v. 5.10.1 (Euformatics Oy, Espoo, Finland) or BWA-mem and rescued using Samtools alignment/Map (Hinxton, UK) (v. 1.9).

SARS-CoV-2 sequences were classified using the Pangolin COVID-19 Lineage Assigner tool v. 3.1.5 (https://pangolin.cog-uk.io/) and Nextclade v. 1.4.0 (https://clades.nextstrain.org/). Mutations were identified using Nextclade.

The table shows the analyzed data stratified according to different methodologies used for the variant monitoring and months of sampling collection.

**Fig. 1:**
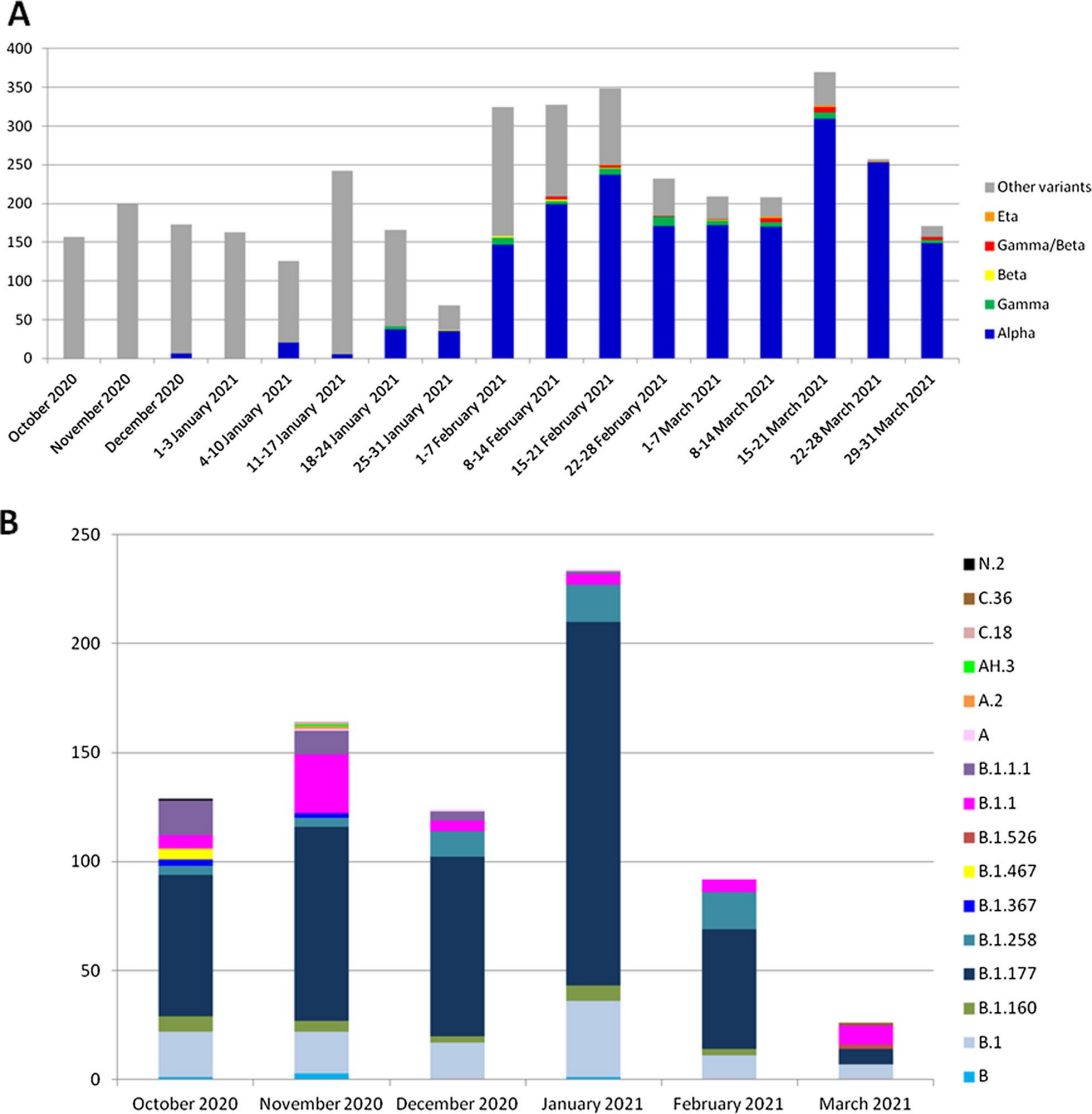
SARS-CoV-2 viral variants observed overtime in Italy. **A** highlights the variants of concern; **B** shows all other circulating lineages

The number of samples considered for variant monitoring increased from 4.2% (158/3744) in October to 32% (1215/3744) in March, due to the increasing interest in viral variants at global level. In the study period, the Alpha variant (B.1.1.7) significantly increased, growing from 3.5% (6/173) in December, to 86.7% (1054/1215) in March (*p* < 0.001). Particularly, from the second half of December this lineage was already present in Liguria (n = 1 on the 18th), Campania (n = 3, on the 28th) and Marche (n = 2, on the 31st) (EPI_ISL_778869, EPI_ISL_778868), with only one known case related to return from UK. The most important increase in its prevalence was observed from the second week of January (20/126, 16.0%) to the end of the same month (35/68, 51.5%) reaching 73.7% (171/232) at the end of February. In parallel, the prevalence of previously circulating variants significantly decreased from 100% (158/158) to 9.4% (114/1215). The Gamma variant (P.1) was first detected in the second half of January in Campania (n = 1), Lombardy (n = 1) and Umbria (n = 2), subsequently setting at around 2% in February (33/1234) and March (24/1215). Only few Beta and Eta variants (B.1.351 and B.1.525 respectively) cases were identified, never reaching 1% of the total samples analysed, being firstly detected in Liguria at the end of January and in Campania in mid-February, respectively. The B.1.258 lineage (also known as Scottish variant) was firstly observed in Campania, Marche and Piedmont in mid-October, 4 cases were reported in November in Campania (n = 2) and Piedmont (n = 2), thereafter it was only reported in Campania until February reaching a proportion of 18.5% (17/92) of total cases of other variants, including its descendant lineages (B.1.258.3, B.1.258.14). In half of March, two cases of the Iota variant (formally known as New York lineage B.1.526) were identified in Marche. Figure 1 shows the main viral variants observed overtime. Concerning other variants, the main circulating lineage was B.1.177 that represented, together with its descendents (B.1.177.4, B.1.177.8, B.1.177.14, B.1.177.15, B.1.177.52, B.1.177.53, B.1.177.75), more than half of cases (50.4%, 54.3%, 66.1%, 71.4% and 59.8% from October to February). Despite limited data regarding lineage assignment in March (n = 28), we observed a 27% prevalence of B.1 and B.1.177 lineages (Fig. 1, Panel B). Clade assignment was available for a larger number of strains compared to lineage because these data was obtained also by spike sequencing highlighting the clear predominance of 20E(EU1) ranging from 43.2% (68/157) to 67.7% (189/279). Some noteworthy mutations were observed in different lineages: N439K in one lineage A and B.1, E484K in three B.1.177 and A222V + E484K + N501Y in clade 20E.

## Conclusion

This study provides insights into the rapid change in the prevalence of SARS-CoV-2 variants over six months in Italy. Interestingly, we observed the first entry of Alpha variant in Lombardy in late December, in line with the identification of first retrospective data in UK on 20 September [9–11] and those reported by ISS-Istituto Superiore di Sanità (https://www.iss.it/documents/20126/0/11-6-2021+SECONDO+BOLLETTINO+Varianti+SARS-CoV-2.pdf/7febfab3-c32b-7505-cc1e-2176fc12cdf6?t=1623430432808). The introduction of Alpha variant, notoriously associated to higher transmissibility compared to other variants and to an increased rate of mortality reported in some studies, corresponds to the second relevant increase of infections registered in Italy during the second epidemic wave [12–14].

In addition to known variants of concern, we documented other minor variants that could be early warnings of upcoming changes. Continuous monitoring of all variants is mandatory to comprehensively investigate and keep track of virus evolution, particularly along with expanding vaccination still based on original strains that have been largely substituted by novel variants.

## Data Availability

The datasets used and/or analysed during the current study are available from the corresponding author on reasonable request. All consensus genomes are being submitted at the GISAID database (https://www.gisaid.org).

## Abbreviations

SARS-CoV-2: Severe Acute Respiratory Syndrome Coronavirus 2
RBD: Receptor-binding domain
RT-PCR: Real time polymerase chain reaction
WGS: Whole genome sequencing
